# Spatial Evaluation of Heavy Metals Concentrations in the Surface Sediment of Taihu Lake

**DOI:** 10.3390/ijerph121214966

**Published:** 2015-11-27

**Authors:** Yong Niu, Wei Jiao, Hui Yu, Yuan Niu, Yong Pang, Xiangyang Xu, Xiaochun Guo

**Affiliations:** 1 College of Hydrology and Water Resources, Hohai University, Nanjing 210098, China; ny0626@sina.com (Y.N.); pangyhhu@126.com (Y.P.); xiangyanghhu@126.com (X.X.); 2 Research Center of Lake Environment, Chinese Research Academy of Environmental Science, Beijing 100012, China; guoxiaochun419@163.com; 3 School of Environment, Beijing Normal University, Beijing 100875, China; weijiao626@126.com

**Keywords:** heavy metals, sediment, spatial distribution, toxic unit, Taihu Lake

## Abstract

With regard to the size of China’s freshwater lakes, Taihu Lake ranks third and it plays an important role in the supply of drinking water, flood prevention, farming and navigation, as well as in the travelling industry. The problem of environmental pollution has attracted widespread attention in recent years. In order to understand the levels, distribution and sources of heavy metals in sediments of Taihu Lake, random selection was carried out to obtain 59 samples of surface sediment from the entire lake and study the concentrations of Pb, Cd, Cu, Zn, Cr and Ni. Toxic units were also calculated to normalize the toxicities caused by various heavy metals. As a result, Cd and Cu in sediment were considered lower than the effect range low (ERL) at all regions where samples were gathered, while Pb and Ni were categorized into ERL-effect range median (ERM) at over 22% of the regions where samples were obtained. Nevertheless, all average concentrations of the samples were below the level of potential effect. According to the findings of this research, significant spatial heterogeneity existed in the above heavy metals. In conclusion, the distribution areas of heavy metals with higher concentrations were mainly the north bays, namely Zhushan Bay, Meiliang Bay as well as Gonghu Bay. The distribution areas of Cu, Zn, Cr and Ni with higher concentration also included the lake’s central region, whereas the uniform distribution areas of those with lower concentrations were the lake’s southeast region. In addition, it was most probable that the spatial distribution of heavy metals was determined by river inputs, whereas atmospheric precipitation caused by urban and traffic contamination also exerted considerable effects on the higher concentrations of Pb and Cd. Through evaluating the total amount of toxic units (ΣTU), it was found that higher toxicity existed primarily in the north bays and central region of the lake. If the heavy metals were sorted by the reduction of mean heavy metal toxic units in Taihu Lake in descending order, it would be Pb, Cr, Ni, Cd, Zn and Cu. Generally speaking, these result of analyses are conducive to alleviating the contamination of heavy metals in Taihu Lake.

## 1. Introduction

Heavy metals in the water reservoirs affect environmental quality by accumulating in reservoirs, resulting in serious human health hazards and significant ecological effects throughout the food chain [1,2]. Most heavy metals are initially absorbed by the suspended particles in the water, after which they undergo a series of processes and are then deposited into the sediment [3,4]. When external environmental factors such as pH, Eh, or temperature change, metals in the sediment can be released back into the water column causing secondary pollution [2]. Thus, it is essential to attach great importance to the contamination of heavy metals during the strategy making of water management.

Due to the fact that the levels of heavy metals in aquatic environment are not the same in different seasons or other environmental factors, the contamination status cannot be indicated by heavy metals all the time [3]. As the sediment cannot only carry but also absorb different kinds of pollutants in water, they can be regarded as a helpful indicator of contamination [4]. Nevertheless, because of some certain disturbances, it is still possible for the heavy metals to be released back to the upper water, which may lead to the serious destruction of the ecological systems [5]. Therefore, analyzing and evaluating the sediment is of great significance in assessing the contamination status of heavy metals in water ecological environment. At present, distinct organizations in both federal and provincial levels have put forward the numerical sediment quality guidelines (SQGs for short), which is conducive to the evaluation of environmental threats caused by heavy metals [6].

It was reported that among 27 lakes monitored in China, almost 60% of them have been polluted by anthropogenic activities and could not have met the requirements of daily life and agricultural irrigation [7]. Taihu Lake is one of the lakes to which great importance has been attached by the Chinese government in the control of water contamination, and its serious eutrophication is well known to the public [8,9]. Nevertheless, with the constant increase of population and rapid growth of economy in its surrounding regions, this lake is also faced with a serious problem of heavy metal contamination. It can be seen from the research of Fu *et al.* [10] that a much higher degree of accumulation of heavy metals has been seen in fishes from the Taihu Lake compared with those from the Yangtze River, which will exert negative influence on human health. Furthermore, in order to further supervise the contamination in Lake Taihu basin, an extensive analysis has been carried out in the sampling gathered from Taihu Lake as well as its significant tributaries [11,12,13]. Nevertheless, these investigations focused primarily on natural waters and were generally focused on the small scale. Fewer studies have paid attention to how heavy metals are distributed in sediment and the evaluation of threats in the scale of the entire lake. 

Thus, following major targets are set for this research: firstly, it aims to examine how Pb along with Cd, Cu, Zn, Cr and Ni distributes in the overlying deposits of Lake Taihu; secondly, it aims to find out the source of heavy metal contamination through multivariate analysis; and thirdly, it aims to evaluate the environmental threats of these heavy metals based on the SQGs and the total amount of toxic units, thereby enabling ranking and prioritization of sites and metals of concern.

## 2. Materials and Methods

### 2.1. Description of Research Area

**Figure 1:**
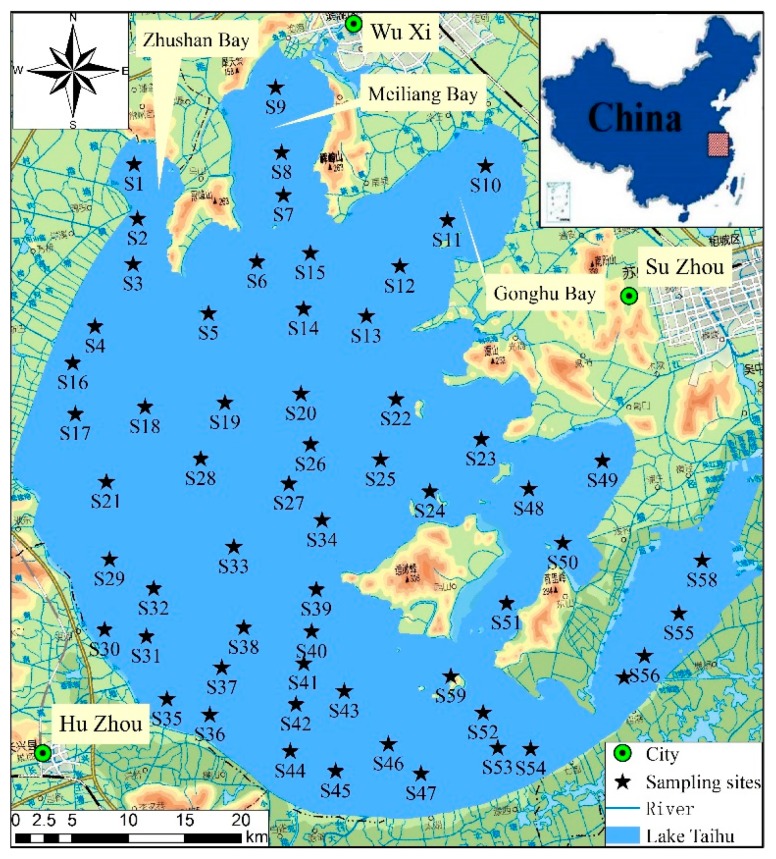
Location of sampling sites in the Lake Taihu, Jiangsu Province, China.

With regard to the size of China’s freshwater lakes, Taihu Lake, situated in lower Yangtze River Delta between 30°56″–31°33′ N and 119°53′–120°36″ E as shown in Figure 1, ranks third. Despite of its total surface area of 2338 km^2^, Taihu Lake has a mean water depth of only 1.9 m [14]. At the same time, this lake is regarded as a vital source of drinking water for a few of cities like Shanghai as well as Suzhou and in general the feature of Taihu Lake basin can be concluded as a great plain of river networks because about 200 rivers as well as tributaries flow into it. Due to the fact that the surrounding areas of Taihu Lake experience a rapid economic growth, serious water contamination has been experienced in this lake from the 1980s [8]. Nowadays, according to the Chinese Environmental Quality Standards for Surface Water (GB3838-2002), water in Taihu Lake is lower than the standard of Class V.

### 2.2. Collection and Analysis of Samples

In August of 2013, researchers gathered 59 samples of surface sediment at random from the entire Taihu Lake as shown in Figure 1. At every place where the samples were obtained, a Petersen grab of stainless steel material was used to take and intensively mix three sub-samples for purpose of acquiring the composite sample. Then the samples were taken to the laboratory where they experienced air drying at ambient temperature and grind with the application of petal and mortar so that a nylon filter of 0.149 mm could be passed through by them. In order to investigate the concentration of Pb, Cd, Cu, Zn, Cr as well as Ni, the approach of HNO_3_–HF–HClO_4_ as well as inductively coupled plasma-atomic emission spectroscopy (ICP-AES) was applied to the digestion and determination of the samples that had been dried. In addition, an automatic analyzer (Shimadzu TOC 5000) was adopted to identify the concentration of total organic carbon and the reference material GBW-07402 was also used to assess the quality of analytical data at the same time. Consequently, great consistency was achieved between the values that have been measured and certified, while the recoveries were in the range between 96.74% and 105.22%, Pb 96.74%, Cd 105.22%, Cu 99.21%, Zn 98.46%, Ni 103.16%, Cr 97.74% individually.

### 2.3. Sediment Quality Guidelines

Sediment quality guidelines (SQGs) have been developed by various federal and provincial agencies in North America for both freshwater and marine ecosystems, which are very useful to screen sediment contamination by comparing sediment contaminant concentration with the corresponding quality guidelines [10]. These guidelines assess the degree to which the sediment chemical status might adversely affect aquatic organisms and are designed to assist the interpretation of sediment quality. With regard to the assessment of environmental threats resulted from heavy metals in sediment, two sets of SQGs were adopted, namely the threshold effect level (TEL)/probable effect level (PEL) and the effect range low (ERL)/effect range median (ERM) values [15,16]. ERLs and TELs are categorized into the low range values referring to the concentrations below which adverse effects upon sediment dwelling fauna will be infrequently. Nevertheless, ERMs along with PELs stand for the concentrations above which negative influences are likely to occur [15]. Besides, toxic units (TU) were also calculated to normalize the toxicities caused by various heavy metals, which allow the comparison of their relative effects and can be defined as the ratio of the determined concentration (C_i_) to PEL value (P_i_), Equation (1). The sum toxic units (∑TU) are sum of TU_i_ [17].

TU_i_ = C_i_/P_i_ (1)

### 2.4. Spatial and Statistical Analyses

When it comes to the areas where samples were not gathered, it is recognized that Kriging is an effective method of spatial interpolation and forecast [18]. Based on the results of 59 sampling sites, Ordinary Kriging integrated into a Geographical Information System (GIS) was used to obtain the spatial distributions of the potential ecological risk values of heavy metals. On contour maps, distinctly darker shades were used to indicate zones of higher values and lighter shades were used for lower values zones. At the same time, the connection among six heavy metals in the sediment was examined with the application of the Pearson correlation coefficient. In the end, in order to find out probable sources of contamination, factor analysis was carried out through the assessment of major components and calculation of eigenvectors. Besides, the SPSS 17.0 for Windows (Inc. Chicago: Chicago, IL, USA) was applied to all of the statistical analyses conducted in this research.

## 3. Results and Discussion

### 3.1. Average Concentrations of Heavy Metals in Sediment

Table 1 presents the concentrations of six heavy metals including Pb, Cd, Cu, Zn, Cr as well as Ni in the sediment of Taihu Lake. Generally speaking, the concentrations of the above heavy metals fluctuate in a wide range, which can be seen from the fact that Pb, Cd, Cu, Zn, Cr and Ni have the maximum concentrations which are 3.40, 7.64, 3.31, 4.91, 11.61 and 7.25 times larger than their minimum concentrations, respectively. It can be concluded from this result that the concentrations of heavy metals are very different in terms of different areas in the sediment of lakes. The concentrations of heavy metal in our study was in order of Zn, Cr, Ni, Pb, Cu and Cd, which was in agreement with the related researches of heavy metals in sediment from Lake Taihu (Table 1). Moreover, all concentrations of Cd, Pb, Zn, Cu and Ni in the sediment were increased and their average concentrations were far greater than the values in basin background. Furthermore, it can be seen from the findings that the average concentration of Pb along with Cd and Cr were greater than the values of TEL. Nevertheless, they were not higher than the values of PEL or ERM. 

**Table 1:** Statistical description of heavy metal concentrations in the surface sediments of Lake Taihu and sediment guideline values (mg/kg).

	Pb	Cd	Cu	Zn	Cr	Ni
Minimum	16.76	0.21	16.1	41.61	12.08	10.87
Maximum	57.09	1.61	53.24	204.37	140.22	78.83
Average	29.87	0.74	28.27	79.74	72.11	34.33
S.D.	8.27	0.23	6.11	26.89	29.48	13.93
CV (%)	28%	31%	22%	36%	42%	41%
TEL ^a^	18	0.596	35.7	123	37.3	35
PEL ^a^	36	3.53	197	315	90	91.3
ERL ^a^	35	5	70	120	80	30
ERM ^a^	110	9	390	270	145	50
Yin [19]	51.8	0.94	36.7	/	56.2	/
Yuan [20]	36.3	/	27.2	99.5	86.7	40.6
Fu [10]	33.55	0.14	34.14	105.55	68.09	36.23
background value ^b^	15.7	0.27	18.9	59	79.3	19.5

S.D.: standard deviation; CV: coefficients of variation; ^a^ TEL: threshold effect level, PEL: probable effect level [15,16], ERL: effects range low, ERM: effects range median [15,16]; ^b^ Soil background concentrations of heavy metals in Taihu basin [21].

### 3.2. Spatial Distribution of Heavy Metals in Sediment

**Figure 2:**
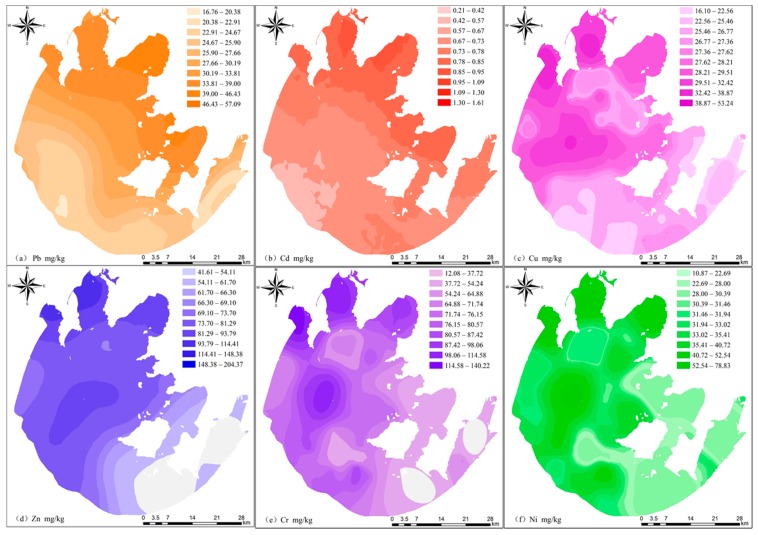
Spatial distributions of (**a**) Pb; (**b**) Cd; (**c**) Cu; (**d**) Zn; (**e**) Cr and (**f**) Ni in the surface sediments of Lake Taihu. Note: On contour maps, distinctly darker shades were used to indicate zones of higher values and lighter shades were used for lower values zones.

With contour maps, Figure 2a–f presents the pattern considering the spatial distribution of the above six heavy metals in sediment. On contour maps, distinctly darker shades were used to indicate zones of higher values and lighter shades were used for lower values zones. According to Figure 2a, the major distribution areas of Pb with higher concentrations were the north bays, namely the Gonghu, Zhuashan as well as Meiliang bays and the eastern lake. The distribution of Cd resembles that of Pb and it was found that the distributions of the above two metals were correlated positively with each other in a significant way, which was shown in Table 2. Despite the fact that not only Cu but also Zn belong to minor nutrients, they may be poisonous for the aquatic organisms if their concentrations are high [22]. Based on Figure 2c,d, the major distributions areas of Cu and Zn with higher concentrations were the north bays and the lake’s central regions, while Figure 2e,f presented the spatial distribution and regional difference of Cr and Ni, which were similar with that of Cu and Zn. In addition, Cr and Ni are often connected with numerous rocks and thus their significant regional difference in soils has been widely recognized even if the scale is small. With regard to this research, all variation coefficients of the above two metals were larger than 40%. Besides, the major distribution areas of Cr and Ni with higher concentrations were the north bays and the lake’s central and southwest regions.

**Table 2:** Correlation matrix between heavy metals in the surface sediments of Lake Taihu.

Element	Pb	Cd	Cu	Zn	Cr	Ni	TOC
Pb	1						
Cd	0.516 **	1					
Cu	0.359 **	0.290 *	1				
Zn	0.344 **	0.319 *	0.760 **	1			
Cr	0.172	0.030	0.551 **	0.678 **	1		
Ni	0.250	0.146	0.630 **	0.753 **	0.932 **	1	
TOC	0.362 **	0.407 **	0.444 **	0.513 **	0.573 **	0.485 **	1

* Represents significant correlation at the level of *p* < 0.05; ** Represents significant correlation at the level of *p* < 0.01.

Based on lots of former researches, because of organic matter’s excellent complexing ability for metallic pollutants, it could play the role of a key sink of heavy metals [7,16]. The total organic matter (TOC) as indicator of organic matter pollution of surface water or sediment was widely accepted and cited by related researches [13,14]. In our study, the TOC varied between 0.76% and 0.23%. The sampling sites with higher concentrations of TOC were mainly distributed in the north bays, the middle and southwest areas, suggesting anthropogenic influence. As shown in Table 2, it was found in this research that the concentrations of heavy metals were positively correlated with those of TOC in a significant way in the sediment, with *p* < 0.01. Furthermore, it was known from the findings of Shi and Sun [23] that considerable contaminants (nutrients, organic matter) could be assimilated by the water plants in relatively shallower water. Mishra *et al.* [24] also reported that aquatic macrophytes revealed high removal (>90%) of heavy metals (Zn, Cu, Cr and Cd) by bioaccumulation. Due to the abundance of plants in the southeastern lake, the concentrations of heavy metals in the sediment may be lower and reducing the internal contamination through plant harvest may be an effective method.

### 3.3. Source Identification of Heavy Metals in the Sediment

Important information about the source of contaminants can be offered by the correlation among different heavy metals. It can be seen from Table 2 that Pb and Cd were positively correlated with each other in a significant way. However, none of them had presented significant correlations with Cr and Ni, meaning that the input sources of Pb and Cd might be similar, but their sources might differ from those of Cr and Ni. In addition, both Cu and Zn showed a significant correlation with not only Pb and Cd but also Cr and Ni, which indicated that the sources of Cu and Zn might be different.

**Figure 3:**
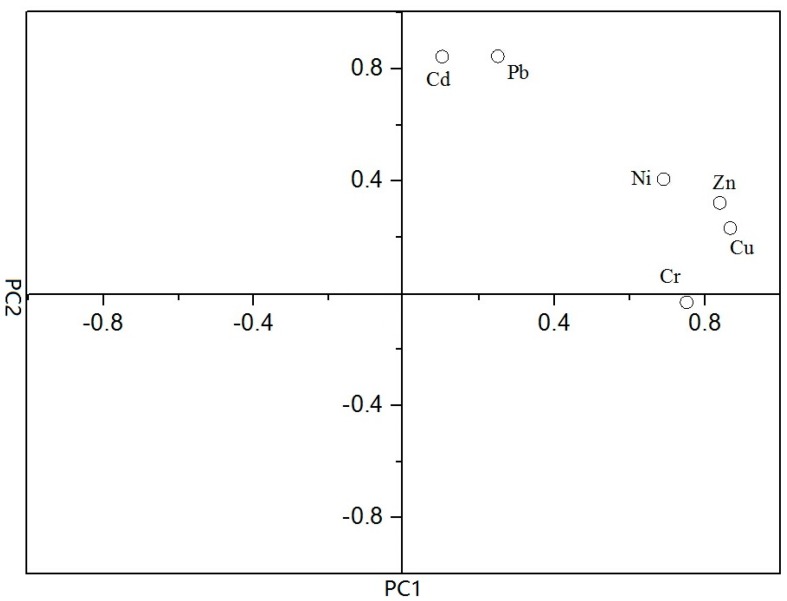
Plot of the two principal components.

The correlations of heavy metals are also likely to be obtained through factor analysis in the factor which will provide some information on the sources of contamination [25]. Table 3 presented the factor analysis results regarding the concentrations of heavy metals in sediment of Taihu Lake. Additionally, a biplot of the PCA loadings is presented in Figure 3. The results of factor analysis for heavy metal concentrations in the surface sediments of Lake Taihu are listed in Table 3. Two factors with eigenvalues greater than 1 were extracted, which explained over 70% of the data variation. The first factor (PC1), with a variance of 51.122%, was strongly and positively correlated with Cu, Zn, Cr and Ni. The second factor (PC2) accounted for 19.606% of the total variance and showed highly positive factor loadings on Pb and Cd. These results suggested that Pb and Cd might come from the common sources, which was consistent with the correlation analysis. According to spatial distribution patterns, Pb and Cd arising from atmospheric deposition might mainly contribute to their accumulation in the north bays and east area, where the urban and traffic pollution was especially serious [26]. By analyzing the soil samples in different historical periods, Huang *et al.* [27] have also demonstrated the impact of atmospheric deposition on Pb and Cd accumulations in this area. However, river inputs most likely controlled the distribution of Cu, Zn, Cr and Ni in the lake sediments [28]. For example, the Cu, Zn, and Ni concentrations in a typical influent river nearby the north bays were 46.5, 149.9 and 58.7 mg/kg, respectively [29]. These values all exceeded their corresponding concentrations in the lake sediments. It should be noted that the patches of higher Cu, Zn, Cr and Ni concentrations also appeared in the middle area of Lake Taihu. This was most likely attributed to the subsequent distribution of river sediments after deposition [30]. In fact, owning to the effect of factors in physical aspect like flat slope or wave, undisturbed sediment are restricted in Taihu Lake. As a result, the sediment in most regions nearby the shores are soft and their thicknesses are different, while the sediment in the lake’s middle area are relatively hard [30].

**Table 3:** Factor analysis of heavy metals in the surface sediments of Lake Taihu.

Initial Eigenvalues				Element	Rotated Component Matrix	
	Total	% of Variance	Cumulative %		PC1	PC2
	Explanation of Total Variance				Component Matrixes	
1	3.067	51.122	51.122	Pb	0.249	0.845
2	1.176	19.606	70.782	Cd	0.101	0.844
3	0.818	13.632	84.361	Cu	0.867	0.232
4	0.396	6.597	90.958	Zn	0.839	0.322
5	0.347	5.780	96.738	Cr	0.751	−0.032
6	0.196	3.262	100.000	Ni	0.690	0.407

### 3.4. Risk Appraisal of Heavy Metals in the Sediment

The above-mentioned SQGs have been extensively applied to the assessment of the extent to which negative influence may be produced by the chemical conditions related to the sediment and such chemical conditions can be adopted to provide help for the improvement of the deposit quality [31]. Meanwhile, the SQGs can also be applied to screening the pollution of sediment by making a comparison between the concentrations of deposit pollutants and relevant quality guidelines related to the ecosystem of freshwater. Based on the viewpoints of Harikumar and Nasir in 2010 [32], a range of minimal influence is represented by the contents lower than ERL which are designed for the evaluation of situations in which little attention is paid to the biological effects. With regard to the contents that are equal to or larger than ERL, but smaller than ERM, a range in which biological effects will be aroused at times is represented by them. A range of possible effect in which negative biological effects can often be aroused is represented by the contents equal to or larger than the values of ERM. Through the comparison between the values of ERL and those of ERM as shown in Table 1, it can be found that the toxicity of sediment from 54 sampling locations was lower because the concentrations of all metals were not larger than the ERMs. However, the toxicity of sediment from the other five sampling locations was higher because the concentration of Ni was larger than the value of ERM. Nickel is essential for animal metabolism, but the excessive ingestion of Ni generates serious toxicological responses, such as vomiting, cramps, convulsions, or even death [33]. Higher concentrations of metals were observed in the north bays (Meiliang bay, Zhushan bay and Gonghu bay), the lake’s central and southwest regions. Therefore, this metal should be given special consideration. At all areas where samples were gathered, the sediment regarding Cd and Cu were considered lower than ERL. Comparatively, the sediment regarding Pb and Cr were categorized into ERL-ERM at over 22% of the areas where samples were obtained. Heavy metals were confirmed to pose potential threats to both aquatic animals [34] and mammals [35,36], as well as to humans [37]. A great deal of research shows that plumbum (Pb) has obvious toxicity effects on organisms, including nerve, blood, growth, gastrointestinal tract and immune disease though inducing oxidative stress and apoptosis [38]. Additionally, as another widespread environmental pollutant, chromium (Cr) was demonstrated to cause tissue injury, including testicular lesions, renal tubular necrosis, liver toxicity and even cancer [39,40].

**Figure 4:**
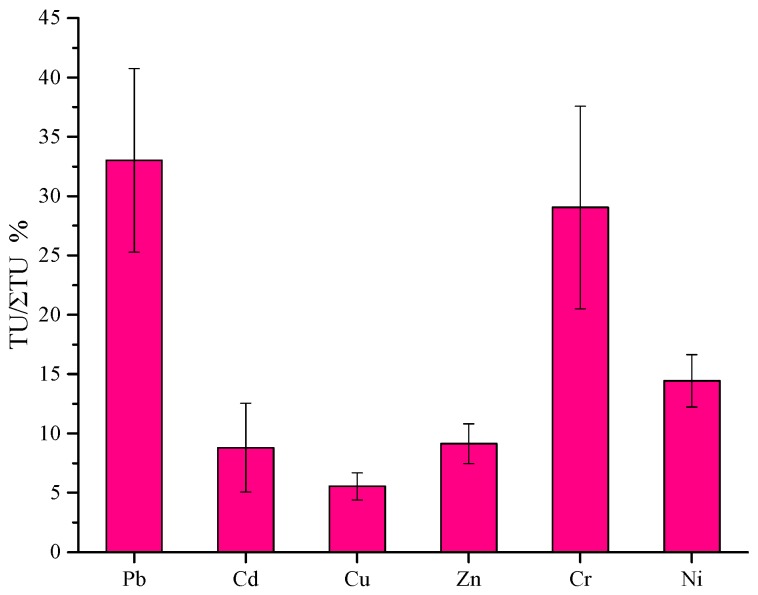
Contributions of respective heavy metal to the sum of toxic units.

Furthermore, for the purpose of assessing the extent to which the aquatic organisms may be influenced by sediment pollution, underlying acute toxicity of pollutants was sincerely suggested. Potential acute toxicity of contaminants in sediment samples can be estimated as the sum of the toxic units (ΣTU) defined as the ratio of the determined concentration to PEL value [17]. In Figure 4, a bar graph presenting the contribution rate of every heavy metal to the total amount of toxic units was shown. The result of sorting the heavy metals by the reduction of mean heavy metal toxic units in Taihu Lake would be: Pb, Cr, Ni, Zn, Cd and Cu, which suggested that the contribution rate of Pb, Cr and Ni to the total amount of toxic units (33.02% ± 7.74%, 29.05% ± 8.53% and 14.44% ± 2.21%, respectively) was higher. Nevertheless, Cu made the lowest contribution to the toxic units, which was 5.54% ± 1.14%.

**Figure 5:**
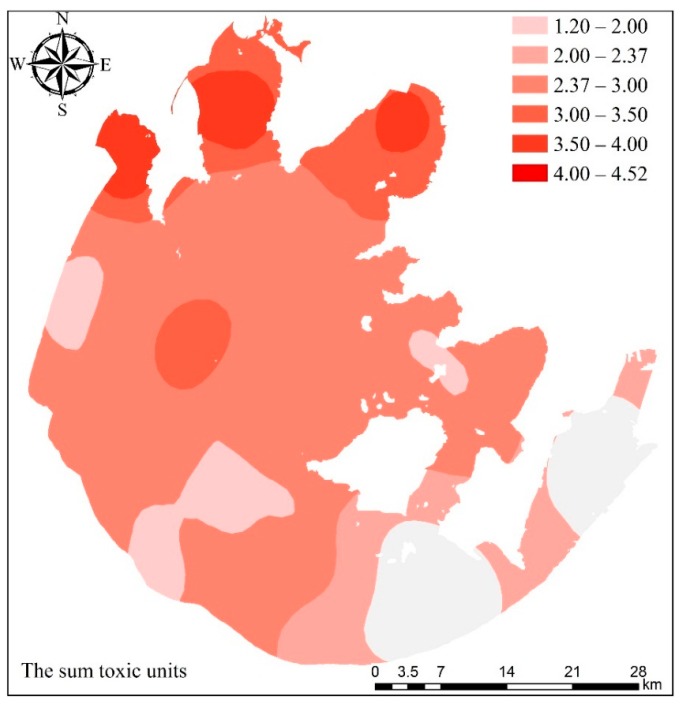
Spatial distribution of the sum of toxic units in the Lake Taihu.

The total amount of toxic units (ΣTU) for the Taihu Lake was computed as per the total amount of the above six heavy metals that had been measured. Subsequently, the contour map indicating the toxic units for the Taihu Lake was drawn and illustrated in Figure 5. All samples with toxic units greater than 4 accounted for over 7% of the total deposit samples, meaning that the toxicity was moderate. According to the contour map, the major distribution areas of sediment with higher toxicity were the regions like the north bays as well as the lake’s central region. Sediments are not only a sink for discharged metals but also a known source of pollutants in lake ecosystem [5]. Heavy metals can be released back to overlying waters from sediments in response to certain disturbances and have potential risks on drinking water source [4]. In addition, aquatic organisms assimilate metals by bio-assimilation and bioaccumulation, with potential long-term implications for human health and the ecosystem [41]. According to a previous study [10], the heavy metals have accumulated in fishes from the Lake Taihu, which were much higher than those from the Yangtze River and might pose risk of adverse health effects to humans. Therefore, it is necessary to control external sources by reducing heavy metal concentrations in industrial sewage discharged into inflowing rivers and remove internal pollution by aquatic plant uptake.

## 4. Conclusions

The major distribution areas of Pb and Cd with higher concentrations were the north bays and east outlet region. At the same time, in the north bays, we observed Cu, Zn as well as Ni with higher concentrations. In comparison, the major distribution areas of the above heavy metals with lower concentrations were the southeast lake, where water plants were prosperous. Besides, it is possible for heavy metals like Cu, Zn, Cr as well as Ni to stem from influent streams; however, atmospheric precipitation caused by urban and traffic contamination played a decisive role in Pb and Cd’s higher concentrations. At present, a threat, namely moderate human-caused contamination, is faced by the Taihu Lake. At all areas where samples were gathered, the sediment regarding Cd and Cu were considered lower than ERL. Comparatively, the sediment regarding Pb and Ni were categorized into ERL-ERM at over 22% of the areas where samples were obtained. Sorting the above heavy metals according to the decrease of their mean heavy metal toxic units in descending order gives: Pb, Cr, Ni, Zn, Cd and Cu. Among the above six heavy metals, Pb, Cr and Ni exerted greater influence on the total toxic units, while Cu exerted the least influence on the toxic units. Therefore, in order to protect the wetland and restore the Taihu Lake in the future, some strategies have to be adopted to decrease the amount of heavy metals in sediment resulting from river input and atmospheric precipitation. Moreover, during the recovery of the ecological system, the introduction of water plants that can grow better in heavy metals is of great significance for the purpose of reducing the ecological threat of Taihu Lake.
